# The role of ultrasonographic findings for *PIK3CA*-mutated, hormone receptor-positive, human epidermal growth factor receptor-2-negative breast cancer

**DOI:** 10.1515/med-2023-0725

**Published:** 2023-11-08

**Authors:** Shuo Li, Qi-Li Zhang, Rui-Jun Guo

**Affiliations:** Departments of Ultrasound Medicine, Beijing Chaoyang Hospital, Capital Medical University, Beijing 100020, China

**Keywords:** ultrasound features, *PIK3CA* mutation, breast cancer

## Abstract

To determine whether ultrasound (US) features of breast cancer are associated with Breast Imaging and Reporting Data System molecular subtype, histologic grade, and hormone receptor status as well as to assess the predictive value of these features. Retrospective analysis of the medical records of 220 consecutive patients with invasive breast cancer was reviewed according to the *PIK3CA*-mutated molecular tumor subtype. US findings of all patients were analyzed. Breast tumors harboring a *PIK3CA*-mutation were large and exhibited liquefied necrosis and posterior echo attenuation in the nodule. Moreover, such tumors were lobulated and calcified. The aspect ratio of the *PIK3CA*-mutant was more likely >1. The average nodule elasticity (7.479 ± 0.993 m/s) was measured using US shear wave elastography. Microcalcification was easier to detect inside the nodule using a fluorescence technique. Measurement of the nodule blood flow spectrum showed that the internal blood flow resistance index of nodules was lower than that of other types of breast cancer. The sonographic features of *PIK3CA*-mutated breast cancers were strongly associated with extensive and liquefied necrosis. The ability to predict molecular subtypes, particularly using US to detect the triple-negative subtype, may play an important role in early management and treatment.

## 1 Introduction

Global cancer data released in 2020 by the World Health Organization International Cancer Research Institute included 2,261,419 new cases of breast cancer and 684,996 [1] deaths; including 416,471 new cases of breast cancer and 117,174 deaths in China (https://gco.iarc.fr/today/home). During the past 20 years, the development of experimental and clinically approved breast cancer-targeting drugs has been progressing rapidly. Examples include endocrine therapy, poly(ADP-ribose) polymerase (PARP) inhibitors (referred to as “PARPi”), phosphatidylinositol-3-kinase PI3K inhibitors, and human epidermal growth factor receptor-2 (HER2) inhibitors, which achieve good therapeutic effects [2–6]. Moreover, despite intensive interest in immunotherapy, it does not effectively treat advanced breast cancer [6,7]. Therefore, targeted therapy continues to play an important role in breast cancer chemotherapy.

The PI3Ka/mammalian target of rapamycin (mTOR) pathway is frequently dysregulated in cancer because of mutation, amplification, or both, of the genes such as those encoding the PI3K catalytic subunits p110a (*PIK3CA*) and p110b (*PIK3CB*), the PI3K regulatory subunit p85a (*PIK3R1*), *AKT1-3*, and the phosphatidylinositol-3,4,5 trisphosphate (*PIP3*) phosphatases *PTEN* and *INPP4B* [7,8]. *PIK3CA* mutations induce a transformed phenotype, including growth factor- and anchorage-independent growth, resistance to anoikis, and drug resistance. Mutations in *PIK3CA* are present in 28–46% of people with hormone receptor (HR)-positive  – HER2-negative advanced breast cancer [8,9].

Alpelisib (BYL719; Novartis Pharma AG, Basel, Switzerland) is an orally administered, selective inhibitor of p110a [3,4]. Notably, more patients still received treatment in the alpelisib plus fulvestrant arm of clinical trials, particularly considering the poor prognosis of these patients with *PIK3CA*-mutated disease who experience shorter survival [6–9].

Ultrasonography is the first choice for screening for breast cancer. The first aim of the ultrasound (US) examination is to identify benign and malignant nodules. Furthermore, with the rise of targeted therapy, it is critically important to identify the characteristics of *PIK3CA* mutations in US images of breast cancer. Therefore, our Radiology Department faces the most urgent key scientific problems such as analyses of the characteristics of US images of different genotypes of breast cancer and implementation of early diagnosis and treatment. This will be of great practical significance to expand the scope of clinical application of drugs and to lengthen patients’ survival rates.

Therefore, the purpose of the present study was to determine whether the US features of breast cancer are associated with Breast Imaging and Reporting Data System (BI-RADS) molecular subtype, histologic grade, and HR status as well as their abilities to predict patients’ outcomes.

## 2 Methods

The US features of 220 consecutive patients with primary invasive breast cancer, who were treated and followed at our breast cancer center between November 2011 and August 2013, were retrospectively evaluated using an electronic database. Of the 220 patients, we excluded 19 with incomplete data and bilateral and recurrent breast cancer. Thirty-three of 201 (16.4%) invasive breast cancers were multifocal, and in these cases, the largest lesion was evaluated. All patients had histologically proven breast cancer and molecular subtypes revealed by analyses of surgical specimens. Patients in the *PIK3CA*-mutant and non-mutant cohorts were randomized.

The Ethics Committee of the Beijing Chaoyang Hospital approved this study (2021-Ke-704). Informed consent from patients with *PIK3CA*-mutated breast cancer was not required by the Ethics Committee for this retrospective study.

### 2.1 Ultrasonography

US scans were performed using a 13–5 MHz linear transducer (VF13–5 Acuson Antares, Siemens, Erlangen, Germany), and the data were evaluated by two radiologists specializing in breast cancer with ≥5 years’ experience performing breast imaging. Both radiologists were uninformed regarding the histopathology results. One radiologist assessed the US images of each tumor from the image archiving and communication system and soft copy images, and the second radiologist was consulted if a case was unclear. All US exams were performed by radiologists, and multiple images were recorded.

The database records of 201 consecutive patients with invasive breast cancer were reviewed according to the molecular subtype of *PIK3CA*-mutated disease. Sonographic tumor sizes were classified as ≤10 mm^3^ and >10 mm^3^; aspect ratios ≤1 and >1; internal echo as low, medium, and high; and ultrasonic shear wave elasticity (USWE) microcalcification as yes or none. Tumor margins were classified as circumscribed or non-circumscribed. The posterior acoustic features were divided into categories as follows: shadowing, enhancement, no change, and mixed pattern. Two senior attending physicians jointly evaluated the images. If there was disagreement, the chief physician was consulted.

### 2.2 Statistical analysis

SPSS 24.0 software (IBM, Chicago, IL, USA) was used for statistical analysis. Normally distributed clinical quantitative parameters are expressed as the mean ± standard deviation and compared using the Student’s *t* test, while non-normally distributed variables are expressed as the median (interquartile range) and analyzed by using the Wilcoxon signed rank test. Data for categorical variables are expressed as numbers (percentages), and the chi-square test was used. Univariate and multivariate logistic regression models were used to analyze the relationship between the classifications. The receiver operating characteristic (ROC) curve was used to determine the optimal cutoff value of the C-statistic that best correlated with the patient’s prognosis. The Kaplan–Meier method was used to estimate overall survival, and *P* < 0.05 indicates a statistically significant difference.

## 3 Results

**Figure 1:**
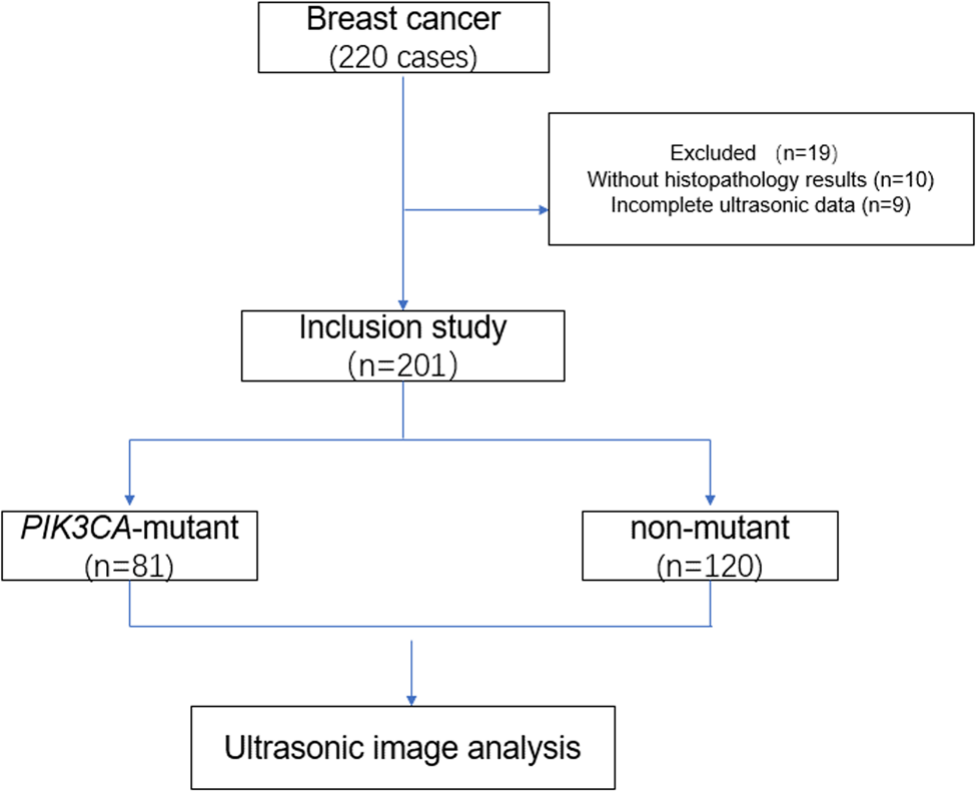
Research flow chart.

Table 1 shows the tumor characteristics and US imaging features of patients included in the study. The mean age at diagnosis of patients with non-mutated tumors was 48 ± 15 years (range, 23–83 years), and the ultrasonographic mean tumor size was 22 ± 14.1 mm. Histologic grade was 1 or 2 in 73 patients (36.3%) and grade 3 in 128 patients (63.7%). Among 201 patients with invasive breast cancer, the molecular subtype was luminal A in 58 (28.9%), luminal B in 99 (49.3%), HER2-positive in 18 (9.0%), and triple-negative in 26 (12.9%). The research flow chart is shown in Figure 1.

**Table 1:** Patients’ tumor characteristics and US imaging features

Contents	Independent variables, *n* (%)			
Dependent variables	*PIK3CA*-mutant (*n* = 81)	Non-mutant (*n* = 120)	*F*	*P*
**Age (years)**			11.733	0.001*
≤50	33	81		
>50	48	39		
**Tumor grade**			118.557	<0.001*
1 or 2	25	110		
3	56	10		
**Sonographic tumor size (mm^3^)**			1.767	0.185
≤10				
>10				
**Margins**			157.757	<0.001*
Circumscribed				
Non-circumscribed				
**Aspect ratio**			117.613	<0.001*
≤1	8	90		
>1	73	30		
**Internal echo (low = 0, medium = 1, high = 2)**			24.465	<0.001*
0	0	52		
1	56	40		
2	25	28		
**Posterior acoustic features**			0.071	0.790
No change				
Enhancement				
Shadowing				
**Microcalcification**			25.214	<0.001*
None	16	63		
Yes	65	57		
**BI-RADS**			0.004	0.948
≤3				
>4				
**USWE**			68.172	<0.001*
*x* ± *s* (m/s)	7.479 ± 0.993	4.435 ± 2.402		

Quantitative data are expressed as the mean ± standard deviation and categorical variables are expressed as number (percentage). *Statistically significant.

### 3.1 US imaging features of *PIK3CA*-mutated tumors

**Figure 2:**
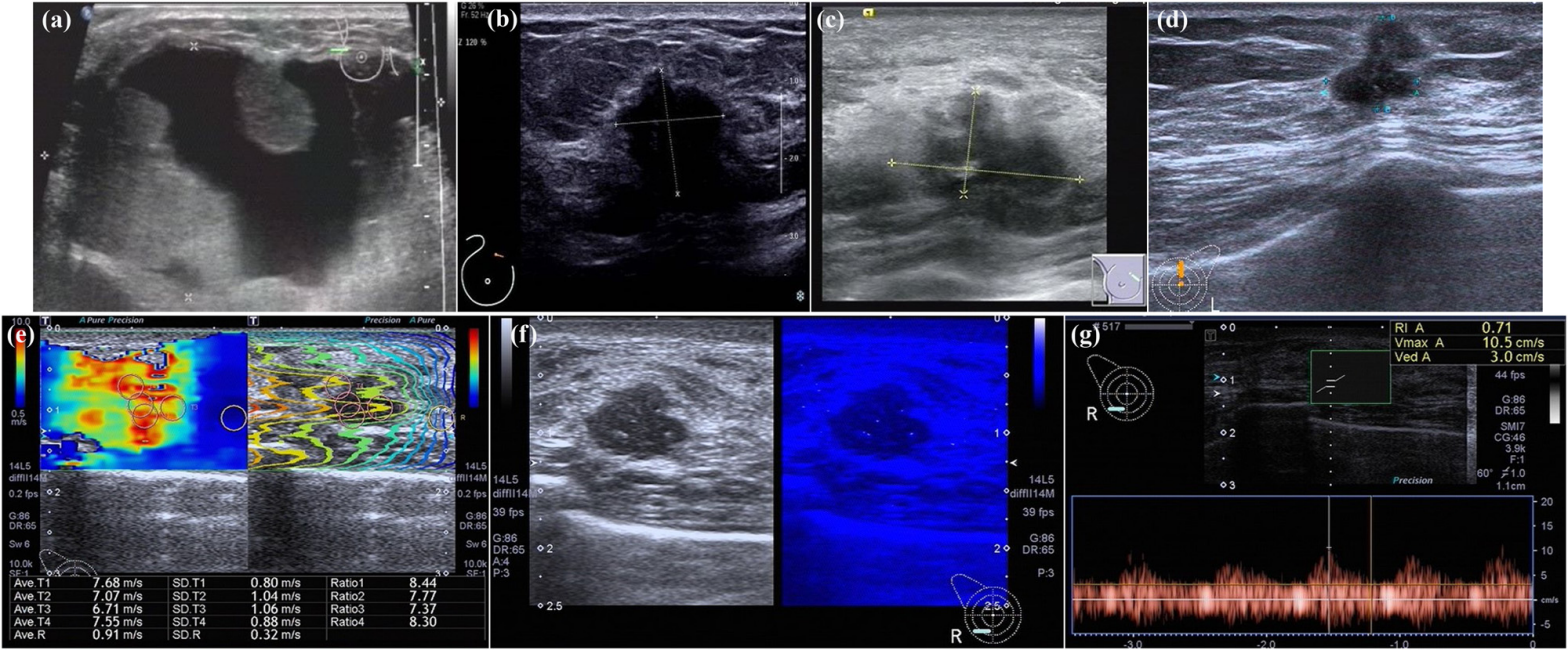
US imaging features of *PIK3CA*-mutated breast tumors. (a) Large nodules with liquefaction necrosis. (b) Echo attenuation behind nodules. (c) Nodules lobulated with inner calcification. (d) Nodule aspect ratio >1. (e) Elasticity measurements of nodules through shear-wave techniques. (f) Intranodal microcalcifications detected using a fluorescence technique (microcalcifications in blue, white light). (g) Nodular blood-flow spectrum measurements show that the intranodular blood flow resistance index is lower than those of other types of breast cancer.

The breast tumors of patients with mutated *PIK3CA* were large and exhibited liquefied necrosis. There was posterior echo attenuation in the nodule, and the tumors were lobulated and calcified. The aspect ratios of *PIK3CA*-mutated tumors were more likely ≥1. The average nodule elasticity was 7.479 ± 0.993 m/s, which was measured using USWE. Microcalcification was more readily detected inside the nodule when a fluorescence technique was employed. Measurement of the nodule blood flow spectrum showed that the internal blood flow resistance index of nodules was lower than that of other types of breast cancers (Figure 2).

We adjusted the factors as follows: age, grade, echoic, margins, aspect ratio, microcalcification, and USWE. Multivariate logistic analysis (Table 2) revealed significant differences associated with age (coefficient: 0.336; 95% CI: 0.253–0.419; *P* < 0.0001), grade (coefficient: 0.281; 95% CI: 0.185–0.376; *P* < 0.0001), echoic (coefficient: 0.113; 95% CI: 0.185–0.376; *P* < 0.0001), margins (coefficient: 0.286; 95% CI: 0.165–0.407, *P* < 0.0001), aspect ratio (coefficient: 0.250; 95% CI: 0.165–0.407; *P* = 0.001), microcalcification (coefficient: 0.147; 95% CI: 0.274–0.020; *P* = 0.024), and USWE (coefficient: 0.051; 95% CI: 0.034–0.069; *P* < 0.0001).

**Table 2:** Logistic analysis results of features of *PIK3CA*-mutated breast tumors

Contents	Coefficients	Std. error	*t* Stat	*P*-value	95% CI
Intercept	0.447	0.063	7.137	0.000	0.571–0.324
Age	0.336	0.042	7.954	0.000	0.253–0.419
Grade	0.281	0.048	5.795	0.000	0.185–0.376
Internal echo (low = 0, medium = 1, high = 2)	0.113	0.029	3.914	0.000	0.056–0.171
Margins (circumscribed = 0, non-circumscribed = 1)	0.286	0.061	4.664	0.000	0.165–0.407
Aspect ratio (0: <1, 1: >1)	0.250	0.078	3.222	0.001	0.097–0.403
Microcalcification (0: none, 1: yes)	0.147	0.064	2.276	0.024	0.274–0.020
USWE	0.051	0.009	5.926	0.000	0.034–0.069

**Figure 3:**
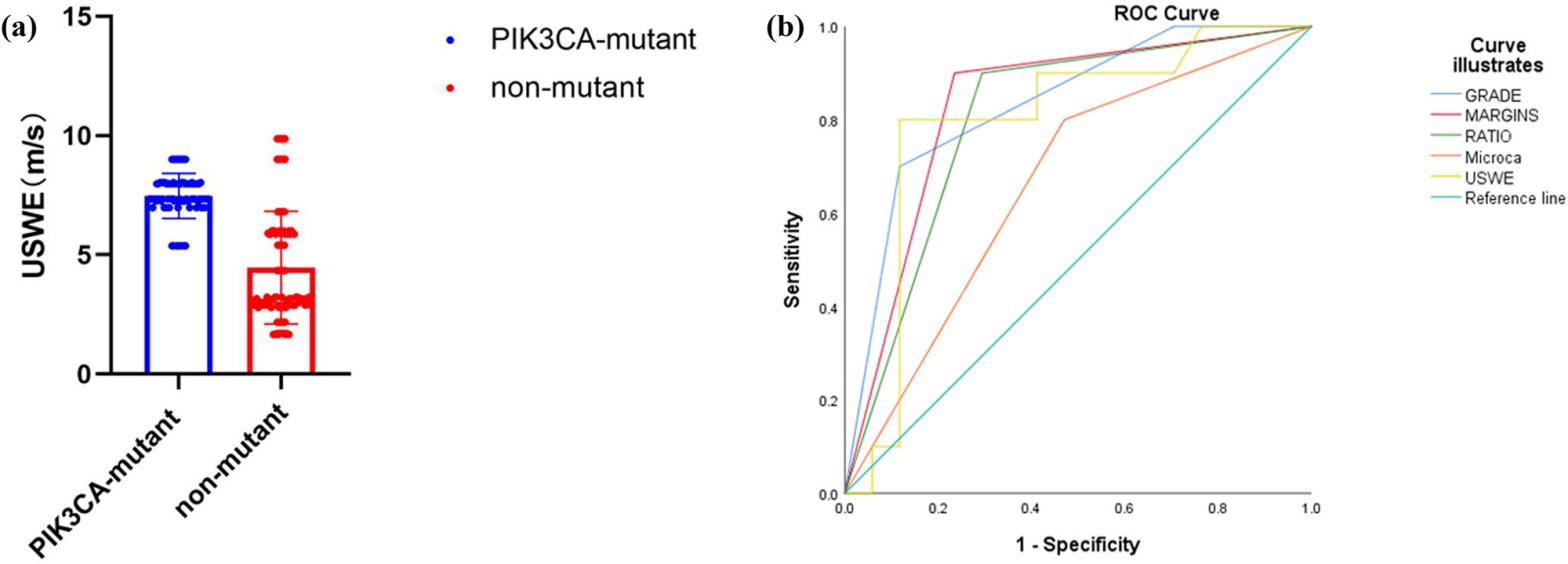
US analysis of *PIK3CA-*mutated and non-mutated tumors. (a) Comparison of USWE between *PIK3CA*-mutated and non-mutated tumors. (b) ROC curve of pathological grade, margin, aspect ratio, microcalcification, and USWE.

**Figure 4:**
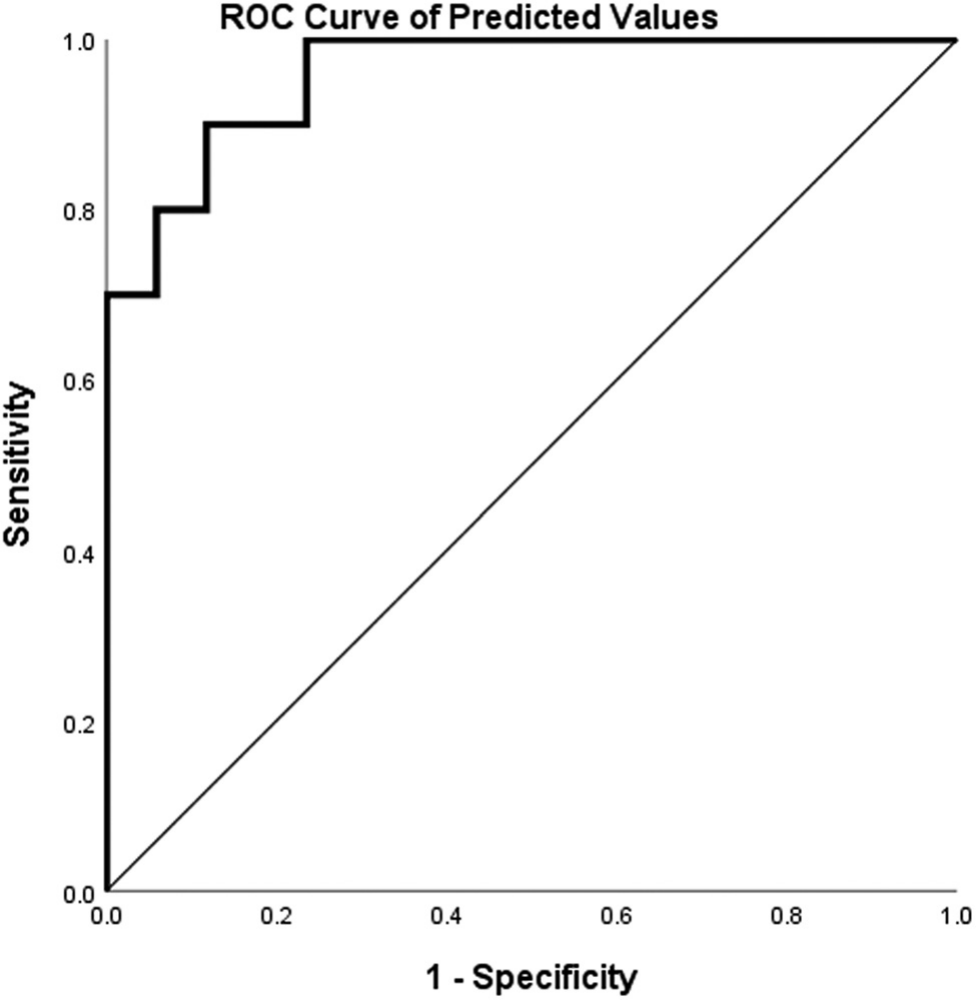
ROC curve-predicted values. AUC: 0.959 (std. error: 0.019; 95% CI: 0.923–0.995; *P* = 0.000).

ROC analysis yielded area under the curve (AUC) values as follows: grade = 0.835 (std. error: 0.01413; 95% CI: 0.9277–0.9831; *P* < 0.0001), margins = 0.832 (std. error: 0.02781; 95% CI: 0.8296–0.938; *P* < 0.0001), aspect ratio = 0.803 (std. error: 0.02726; 95% CI: 0.5193–0.626; *P* = 0.0083), microcalcification = 0.665 (std. error: 0.02683; 95% CI: 0.5610–0.6662; *P* < 0.0001), and USWE = 0.797 (std. error: 0.02381; 95% CI: 0.6851–0.7784; *P* < 0.0001) (Figures 3 and 4).

## 4 Discussion

In the present study, we determined whether the US features of breast cancer were associated with the BI-RADS molecular subtype, histologic grade, and HR status. Furthermore, we assessed the predictive value of these features. Our findings show that the sonographic features of *PIK3CA*-mutated breast tumors were obviously associated with large tumors and liquefied necrosis, suggesting the ability of US to predict molecular subtypes; particularly the triple-negative subtypes may play an important role in early management and treatment.

Breast cancer is a heterogeneous disease with different histopathological and associated biological characteristics [1–3]. Over the past few decades, treatment of breast cancer has entered the molecular, genomics, and proteomics levels, which has improved treatment efficacy to lengthen the survival of patient with breast cancer [4–6]. Previously, breast cancer was treated as a disease without a classification. In 2000, the advent of molecular genetic technology made possible high-resolution molecular typing, culminating in personalized treatment [7–9]. The 70-gene detection MammaPrint microarray was approved for clinical use in China, representing a very important milepost [1,8]. The MammaPrint “70 gene detection” technique provides an important prognostic tool for *ERPR*-positive and *HER2*-negative patients with breast cancer, and the score is calculated at RNA transcriptional level [7–14].

Molecular biological studies reveal the heterogeneity of breast cancer, which help to obtain more precise prognostic and prediction information [10,11]. Therefore, the molecular typing of breast cancer includes the phenotypes as follows: luminal A, luminal B, *Her2* overexpression, and triple-negative breast cancer. Patients in the present study had luminal A and B molecular subtypes (78%).

Breast US has become an effective method to identify benign and malignant lesions, particularly those of young women with dense breast tissue [10–12]. Although many studies focus on the ultrasonographic findings of breast cancer grading, studies on the relationship between tumor subtypes are unavailable [3,8], and imaging data on the identification of molecular subtypes are unavailable as well. Compared with the HR-negative state, HR-positive tumors are more likely to have uncertain margins. In the present study, multivariate analysis revealed significant differences associated with age, grade, echoic, margins, aspect ratio, and microcalcification.

Previously, most malignant breast tumors examined using US were thought to have posterior acoustic shadows with blurry edges [13,14]. However, it is now well known that many tumors may exhibit variable posterior acoustic features. For example, recent studies show that a clear margin and post-enhancement are more likely to represent a higher level of tumor and receptor-negative status [13,15,16]. Our results are consistent with these findings, particularly the statistically significant correlation between a clear margin and post-enhancement.

In the present study, histologic grade was 1 or 2 in 73 patients and grade 3 in 128 patients. Among 201 patients with invasive breast cancer, the molecular subtypes were luminal A in 58, luminal B in 99, *HER2* in 18, and triple-negative in 26. Tumors with HR-positive status (*n* = 154) were more likely to have non-circumscribed margins (*n* = 108), and tumors with HR-negative status (*n* = 47) were more likely to have circumscribed margins (*n* = 32). Circumscribed margins also are among the most frequent alterations in solid tumors, identified in the cancers as follows: 42–55%, endometrial; 24%, cervical; 18%, colorectal, 13%, head and neck; and 12%, ovarian. Targeting the PI3K/mTOR pathway therefore may be particularly effective in cancers that signal through PI3Ka [17–19]. Further studies with larger sample size are needed to investigate the differences in US features among patients with different expression levels of HER2 and HR.

The results of the present study showed that sonographic features of *PIK3CA*-mutated tumors were significantly associated with large and liquefied necrosis. Here we show posterior echo attenuation and microcalcification in the nodule and that the average nodule elasticity was 7.479 ± 0.993 m/s measured using USWE. Furthermore, measurement of the nodule blood flow spectrum reveals a lower internal blood flow resistance index of nodules compared with those of other types of breast cancer. The ability use US to predict molecular subtypes, particularly the triple-negative subtype, may play an important role in optimizing early management and treatment. Large multi-center studies are required to confirm these findings.

## References

[1] Modi S, Saura C, Yamashita T, Park YH, Kim SB, Tamura K, et al. Trastuzumab deruxtecan in previously treated HER2-positive breast cancer. N Engl J Med 382(7):610–21. 10.1056/NEJMoa1914510 PMC7458671 31825192

[2] Turner NC, Ro J, André F, Loi S, Verma S, Iwata H, et al. Palbociclib in hormone-receptor-positive advanced breast cancer. N Engl J Med 373(3):209–19. 10.1056/NEJMoa1505270 26030518

[3] Turner NC, Slamon DJ, Ro J, Bondarenko I, Im SA, Masuda N, et al. Overall survival with palbociclib and fulvestrant in advanced breast cancer. N Engl J Med 379(20):1926–36. 10.1056/NEJMoa1810527 30345905

[4] Robson M, Im SA, Senkus E, Xu B, Domchek SM, Masuda N, et al. Olaparib for metastatic breast cancer in patients with a germline BRCA mutation. N Engl J Med 377(6):523–33. 10.1056/NEJMoa1706450 28578601

[5] André F, Ciruelos E, Rubovszky G, Campone M, Loibl S, Rugo HS, et al. Alpelisib for PIK3CA-mutated, hormone receptor-positive advanced breast cancer. N Engl J Med 380(20):1929–40. 10.1056/NEJMoa1813904 31091374

[6] Baselga J, Campone M, Piccart M, Burris 3rd HA, Rugo HS, Sahmoud T, et al. Everolimus in postmenopausal hormone-receptor-positive advanced breast cancer. N Engl J Med 366(6):520–9. 10.1056/NEJMoa1109653 PMC5705195 22149876

[7] Park JW, Liu MC, Yee D, Yau C, van ’t Veer LJ, Symmans WF, et al. Adaptive randomization of neratinib in early breast cancer. N Engl J Med 375(1):11–22. 10.1056/NEJMoa1513750 PMC5259558 27406346

[8] Schmid P, Adams S, Rugo HS, Schneeweiss A, Barrios CH, Iwata H, et al. Atezolizumab and Nab-Paclitaxel in advanced triple-negative breast cancer. N Engl J Med 379(22):2108–21. 10.1056/NEJMoa1809615 30345906

[9] Schmid P, Cortes J, Pusztai L, McArthur H, Kümmel S, Bergh J, et al. Pembrolizumab for early triple-negative breast cancer. N Engl J Med 382(9):810–21. 10.1056/NEJMoa1910549 32101663

[10] Skaane P, Engedal K. Analysis of sonographic features in the differentiation of fibroadenoma and invasive ductal carcinoma. AJR Am J Roentgenol 170(1):109–14. 10.2214/ajr.170.1.9423610 9423610

[11] Aho M, Irshad A, Ackerman SJ, Lewis M, Leddy R, Pope TL, et al. Correlation of sonographic features of invasive ductal mammary carcinoma with age, tumor grade, and hormone-receptor status. J Clin Ultrasound: JCU 41(1):10–7. 10.1002/jcu.21990 PMC4527309 22996916

[12] Weinstein SP, Conant EF, Mies C, Acs G, Lee S, Sehgal C. Posterior acoustic shadowing in benign breast lesions: sonographic-pathologic correlation. J Ultrasound Med: Off J Am Inst Ultrasound Med 23(1):73–83. 10.7863/jum.2004.23.1.73 14756356

[13] Lamb PM, Perry NM, Vinnicombe SJ, Wells CA. Correlation between ultrasound characteristics, mammographic findings and histological grade in patients with invasive ductal carcinoma of the breast. Clin Radiol. 55(1):40–4. 10.1053/crad.1999.0333 10650109

[14] Shin HJ, Kim HH, Huh MO, Kim MJ, Yi A, Kim H, et al. Correlation between mammographic and sonographic findings and prognostic factors in patients with node-negative invasive breast cancer. Br J Radiol. 84(997):19–30. 10.1259/bjr/92960562 PMC3473801 20682592

[15] Lui VW, Hedberg ML, Li H, Vangara BS, Pendleton K, Zeng Y, et al. Frequent mutation of the PI3K pathway in head and neck cancer defines predictive biomarkers. Cancer Discov. 3(7):761–9. 10.1158/2159-8290.CD-13-0103 PMC3710532 23619167

[16] Vanhaesebroeck B, Guillermet-Guibert J, Graupera M, Bilanges B. The emerging mechanisms of isoform-specific PI3K signalling. Nat Rev Mol Cell Biol. 11(5):329–41. 10.1038/nrm2882 20379207

[17] Nunes-Santos CJ, Uzel G, Rosenzweig SD. PI3K pathway defects leading to immunodeficiency and immune dysregulation. J Allergy Clin Immunol. 143(5):1676–87. 10.1016/j.jaci.2019.03.017 31060715

[18] Hennessy BT, Smith DL, Ram PT, Lu Y, Mills GB. Exploiting the PI3K/AKT pathway for cancer drug discovery. Nat Rev Drug Discov 4(12):988–1004. 10.1038/nrd1902 16341064

[19] Pearson HB, Li J, Meniel VS, Fennell CM, Waring P, Montgomery KG, et al. Identification of PIK3CA mutation as a genetic driver of prostate cancer that cooperates with pten loss to accelerate progression and castration-resistant growth. Cancer Discov 8(6):764–79. 10.1158/2159-8290.CD-17-0867 29581176

